# Supplementary material for: Implications of Error-Prone Long-Read Whole-Genome Shotgun Sequencing on Characterizing Reference Microbiomes
Source: iScience. 2020 Jun 2;23(6):101223. doi: 10.1016/j.isci.2020.101223 (PMC7305381; doi:10.1016/j.isci.2020.101223)
Supplement: Document S1. Transparent Methods, Figures S1–S13, and Tables S1 and S2 [file mmc1.pdf]

iScience, Volume *23*

Supplemental Information

Implications of Error-Prone Long-Read Whole-Genome Shotgun Sequencing on Characterizing Reference Microbiomes

Yu Hu, Li Fang, Christopher Nicholson, and Kai Wang

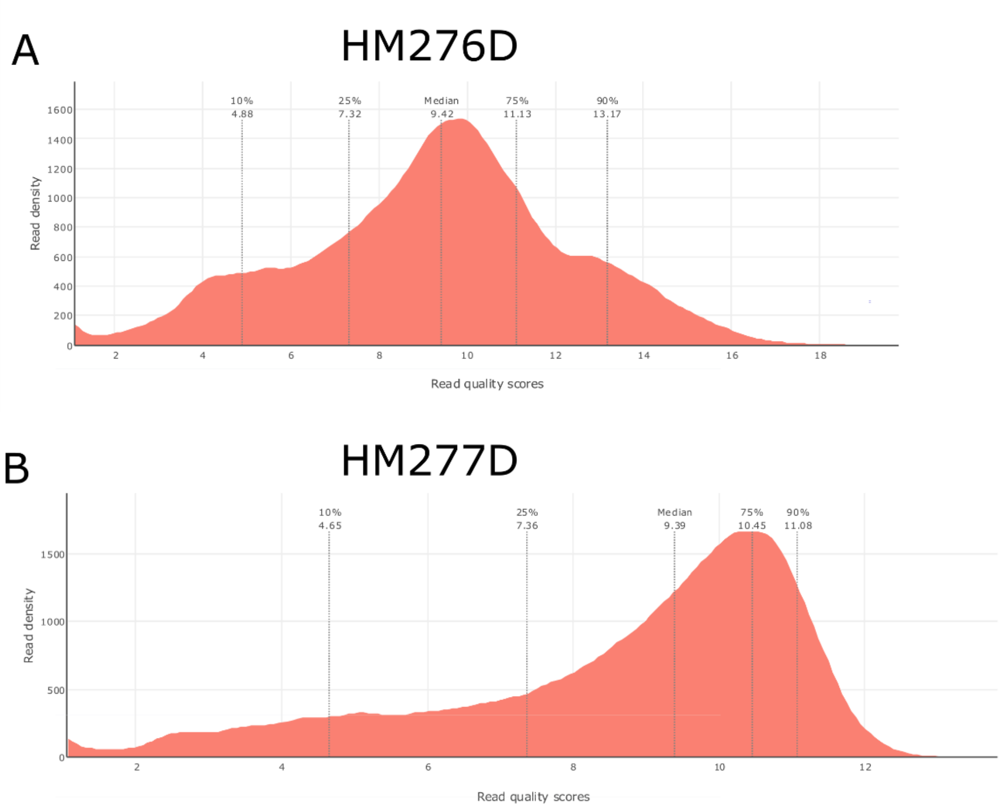

Figure S1. Read quality of Nanopore sequencing data, Related to Table 1. Read quality of sequenced data sets, HM-276D **(A)** and HM-277D **(B)**, were summarized using PycoQC respectively. Dashed lines indicate different quantiles (10%, 25%, 50%, 75%, 90%).

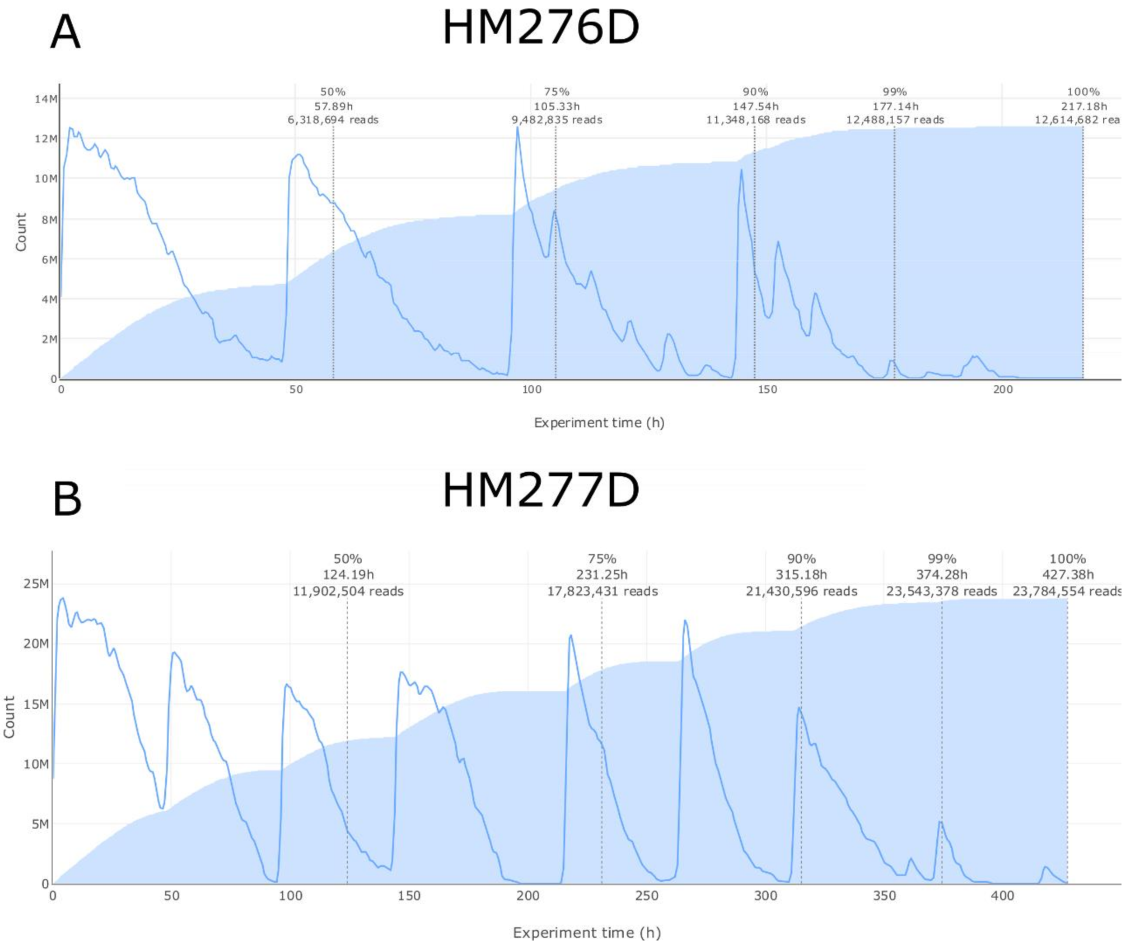

Figure S2. Read output over experiment of Nanopore sequencing data, Related to Table 1. Number of output reads over experiment time for sequenced data sets, HM-276D **(A)** and HM-277D **(B)**, were summarized using PycoQC. Blue line indicates output velocity at specific time. Shaded area represents cumulative read output over experiment time.

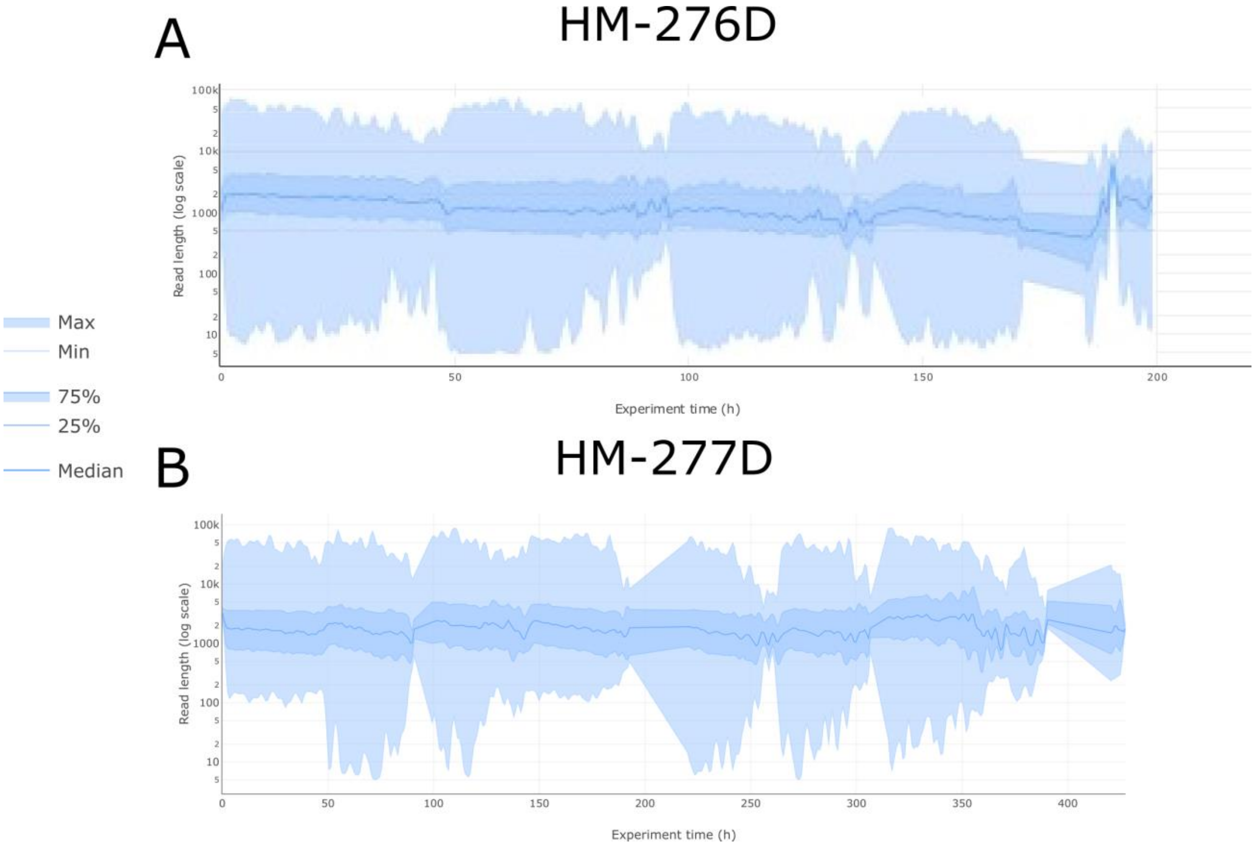

Figure S3. Read length over experiment of Nanopore sequencing data, Related to Table 1. Read length in log scale over experiment time for sequenced data sets, HM-276D **(A)** and HM-277D **(B)**, were summarized using PycoQC.

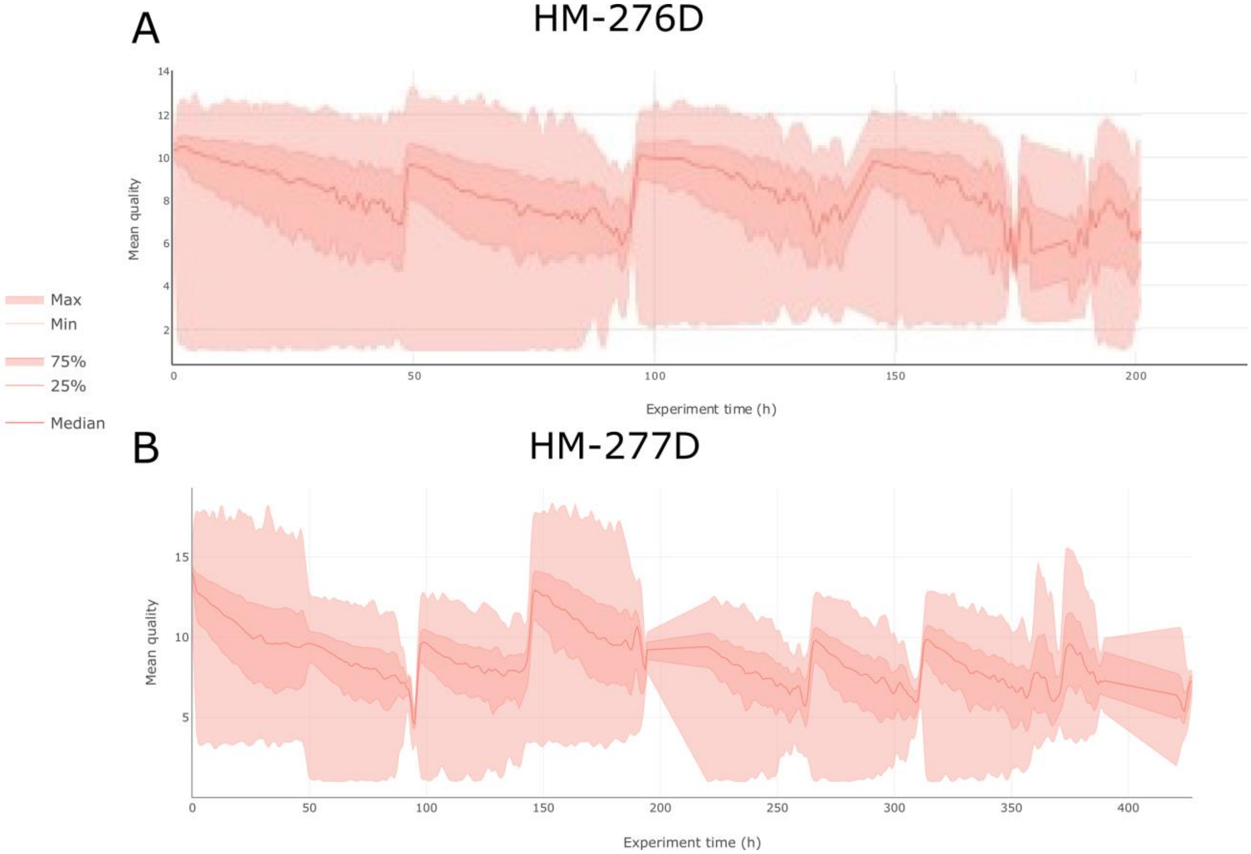

Figure S4. Read quality over experiment of Nanopore sequencing data, Related to Table 1. Mean read quality over experiment time for sequenced data sets, HM-276D **(A)** and HM-277D **(B)**, were summarized using PycoQC.

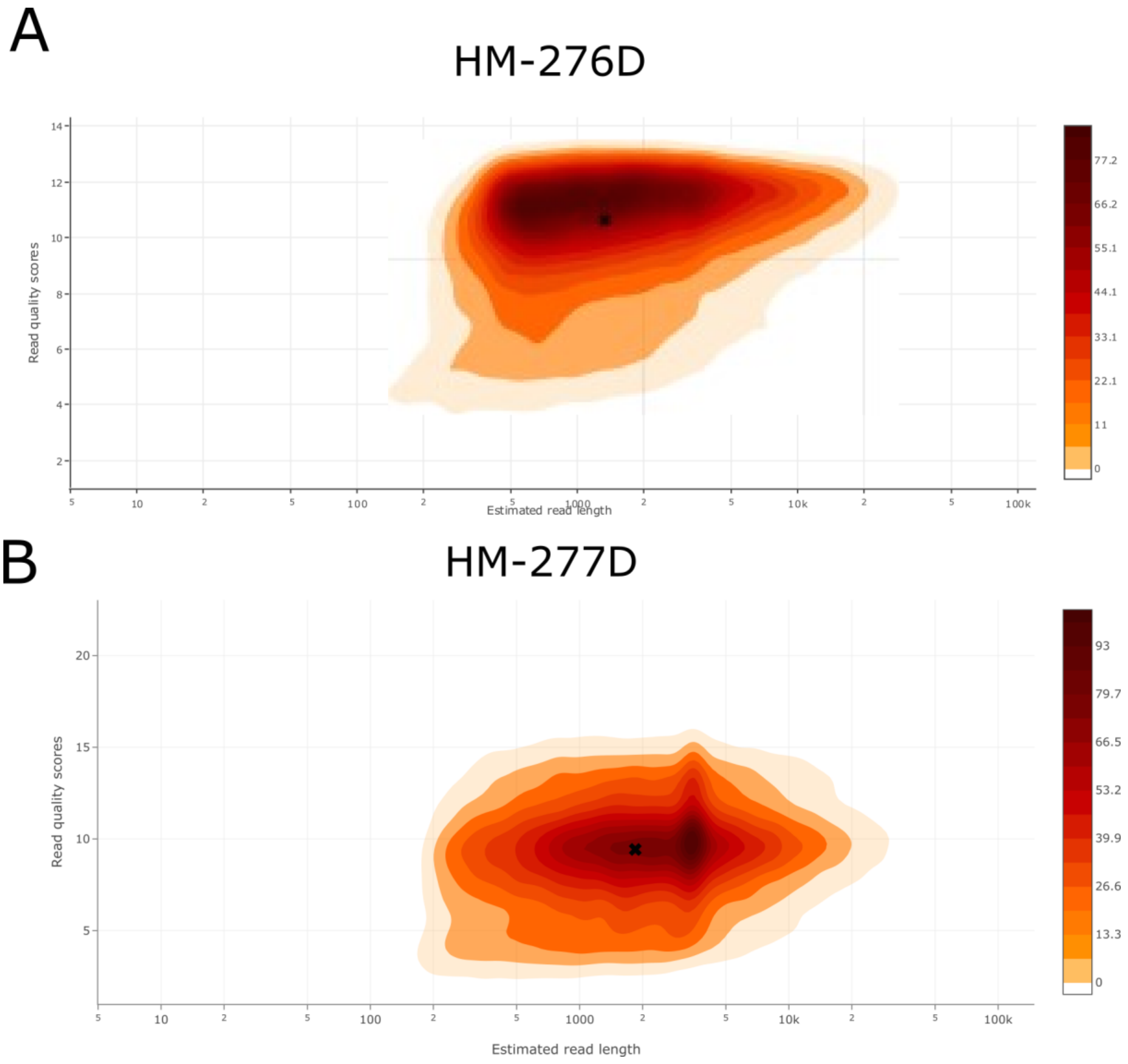

Figure S5. Read quality score vs estimated read length, Related to Table 1. Nanopore read distribution of read length and quality score for sequenced data sets, HM-276D **(A)** and HM-277D **(B)**, were summarized using PycoQC. Color indicates read density.

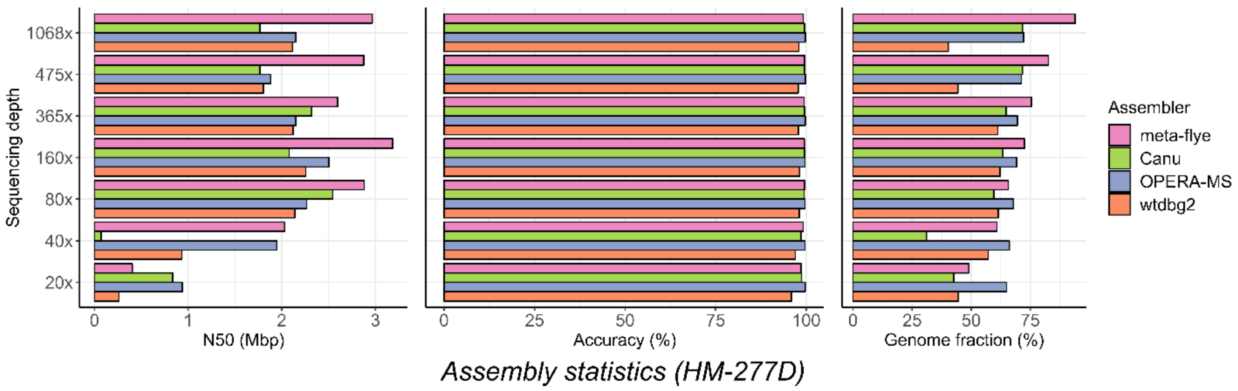

Figure S6. Assembly performance on HM-277D data set, Related to Figure 2. Assembly statistics (N50 length, accuracy and genome fraction) of each assembler at different coverage depths based on HM-277D data set. Colors indicate results from different assemblers (Canu, OPERA-MS, wtdbg2, meta-flye). Assembly accuracy remains high compared to HM-276D, ranging around ~99% across tools. N50 lengths and genome fractions of all methods are substantially lower than the even community.

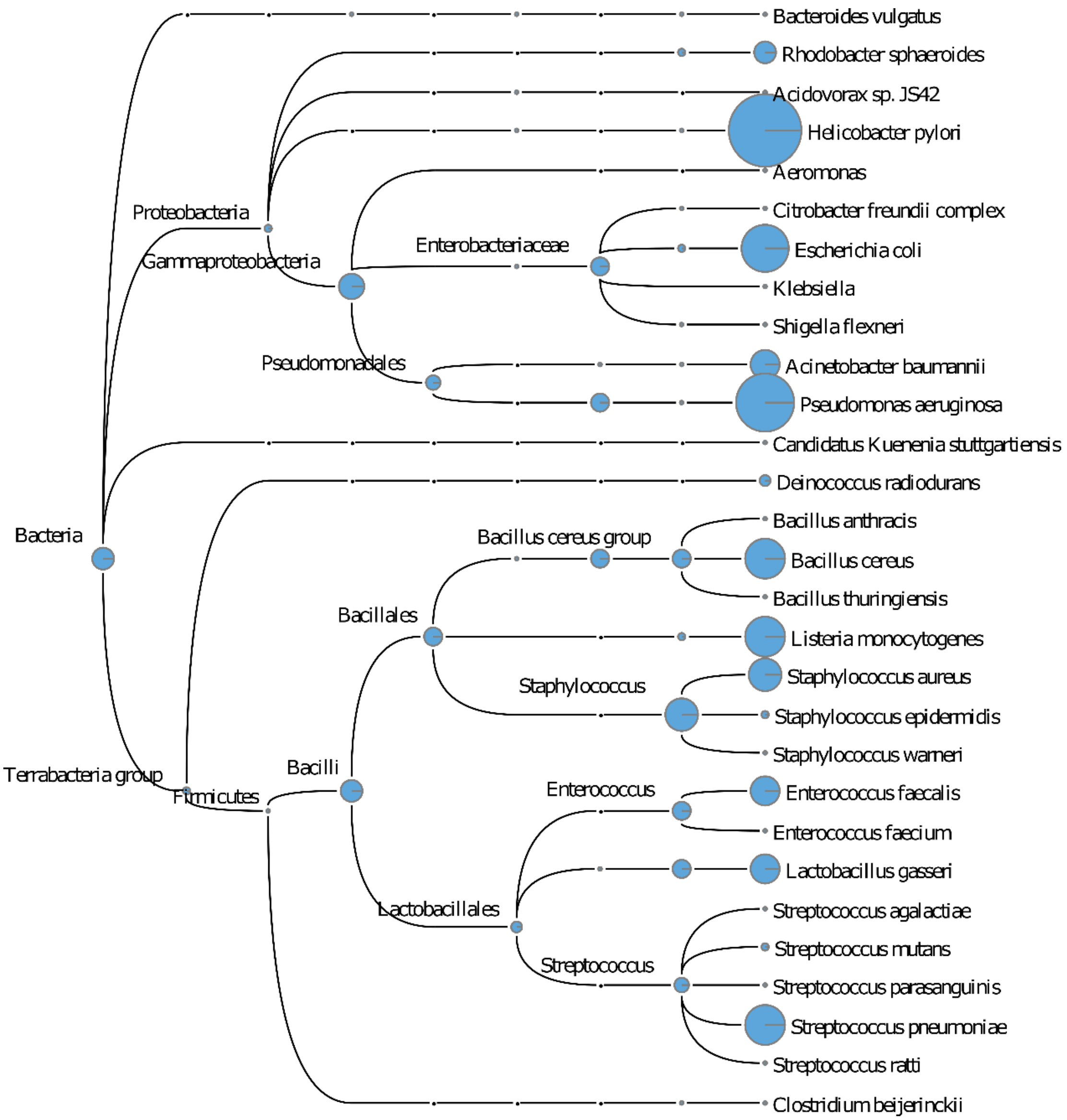

Figure S7. Megan taxonomic tree assignment obtained from HM-276D PacBio sequenced data set, Related to Figure 3. HM-276D PacBio data set was subsampled to 160× depth of coverage. Each read was aligned against NCBI-nr protein reference data base, then binned and visualized using Megan-LR. Megan taxonomic tree showing bacteria taxa identified and their corresponding abundances across taxonomic rank. The radius of circle represents the number of reads assigned for each taxa.

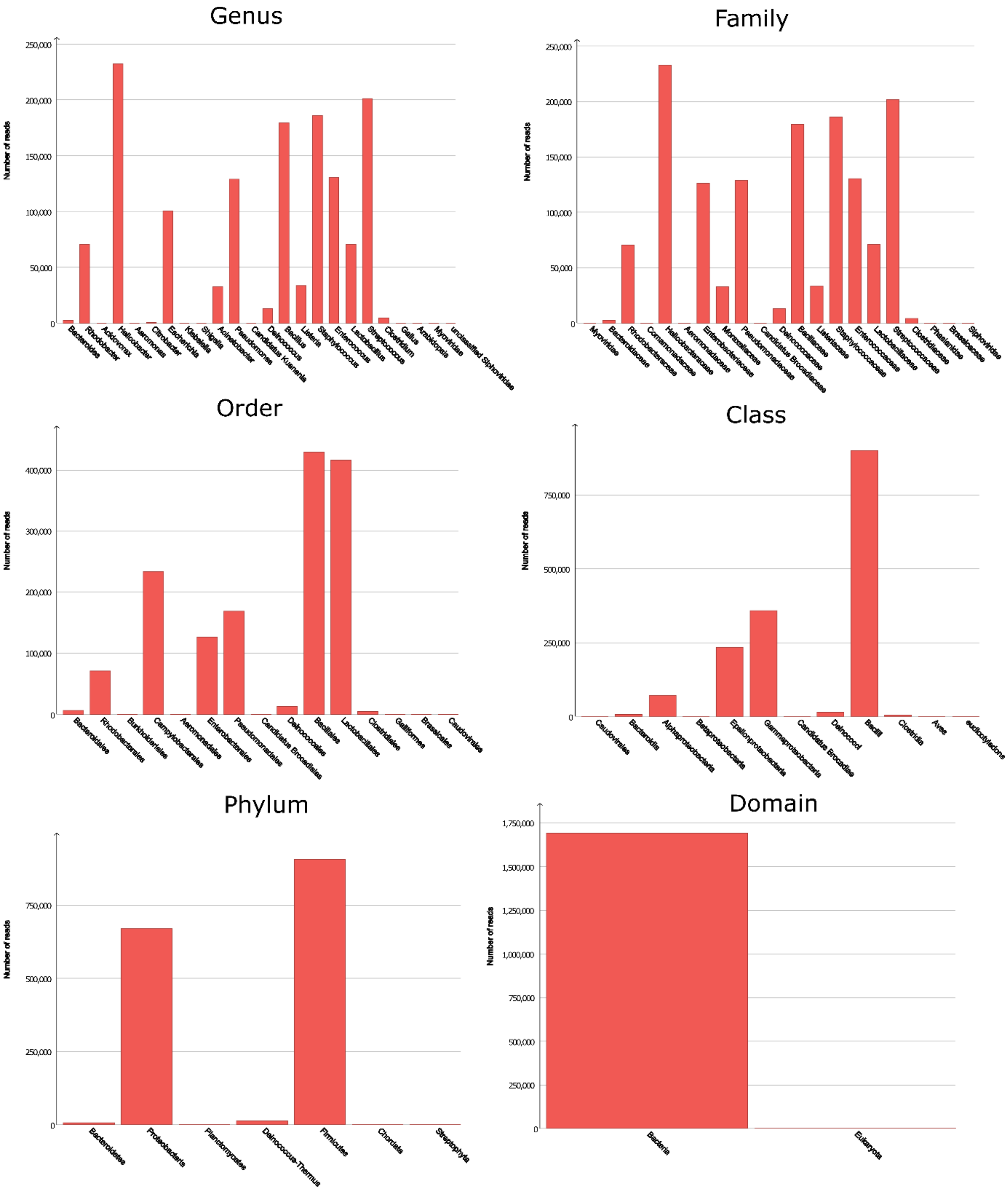

Figure S8. Megan taxonomic read distribution at different ranks obtained from HM-276D Nanopore sequenced data set, Related to Figure 3. HM-276D Nanopore data set was subsampled to 160× depth of coverage. Each read was aligned against NCBI-nr protein reference data base, then binned and visualized using Megan-LR.

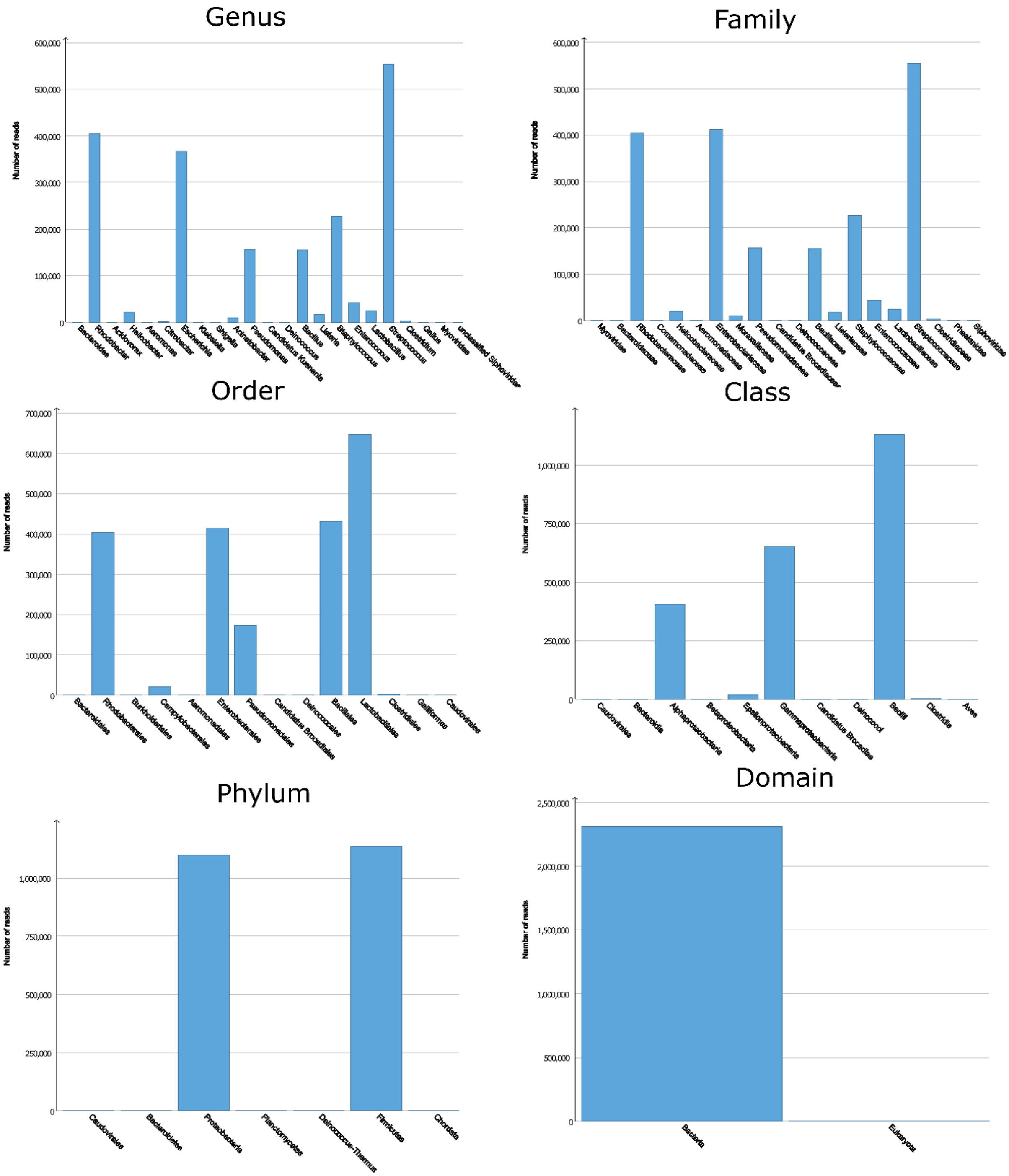

Figure S9. Megan taxonomic read distribution at different ranks obtained from HM-277D Nanopore sequenced data set, Related to Figure 3. HM-277D Nanopore data set was subsampled to 160× depth of coverage. Each read was aligned against NCBI-nr protein reference data base, then binned and visualized using Megan-LR.

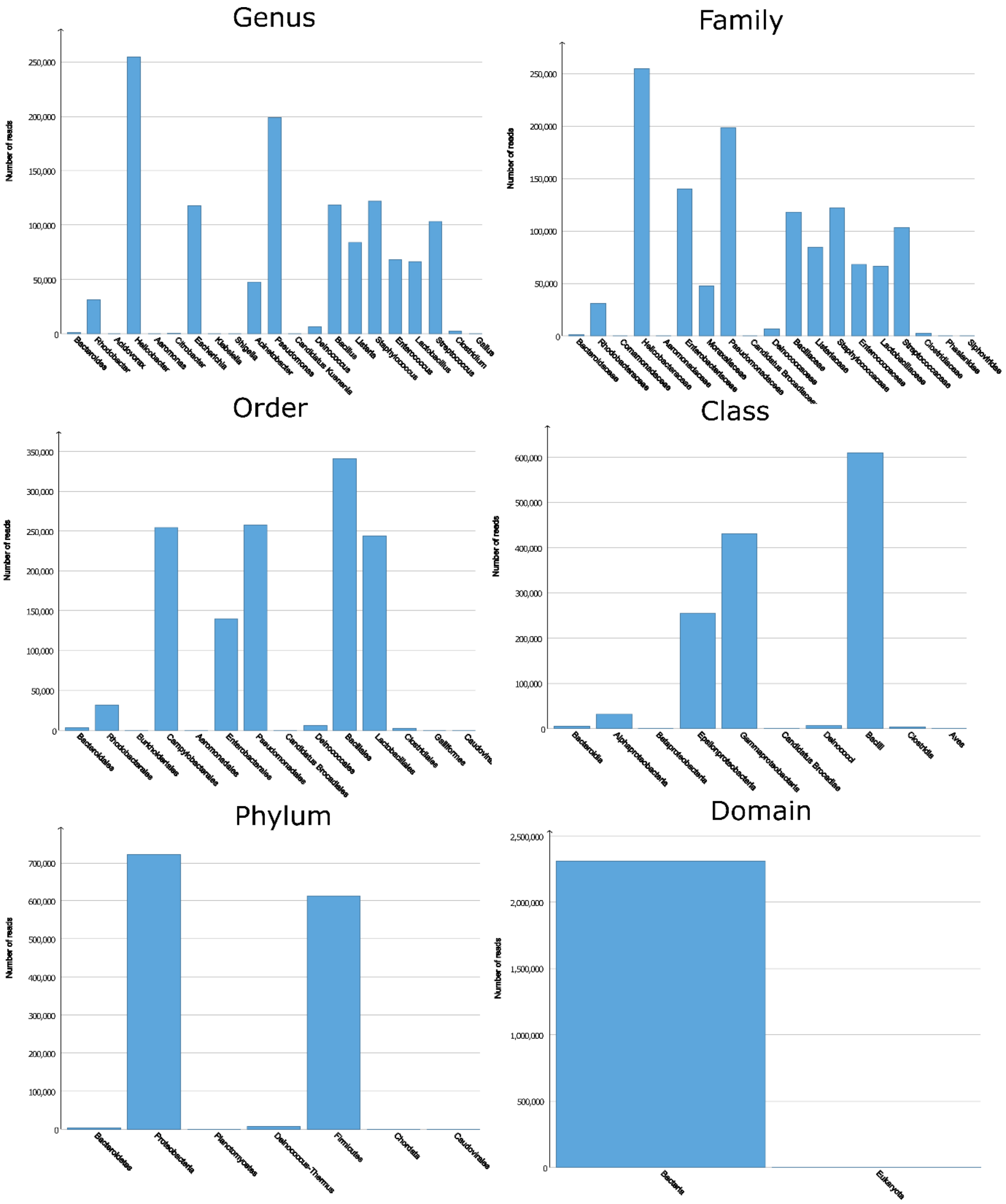

Figure S10. Megan taxonomic read distribution at different ranks obtained from HM-276D PacBio sequenced data set, Related to Figure 3. HM-276D PacBio data set was subsampled to 160× depth of coverage. Each read was aligned against NCBI-nr protein reference data base, then binned and visualized using Megan-LR.

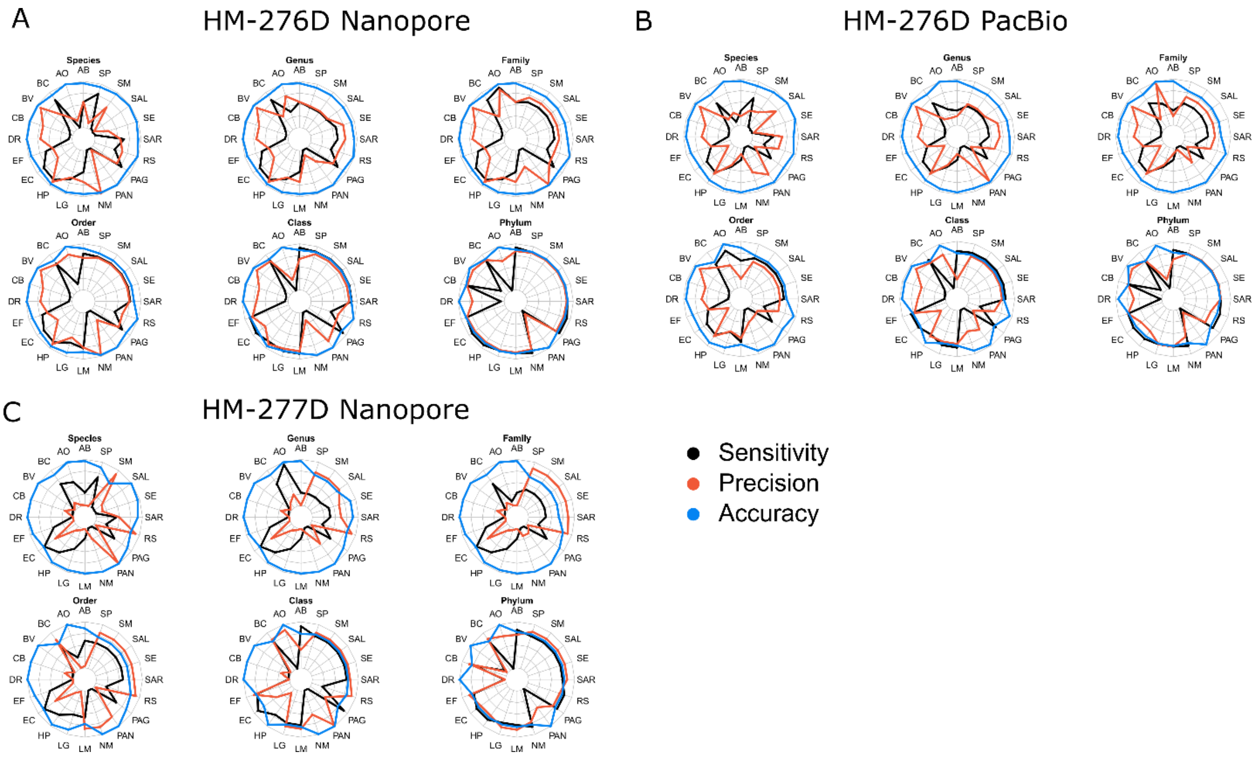

Figure S11. Strain-specific read assignment performance comparison across sequencing technologies, Related to Figure 4. Read assignment accuracy statistics for each bacterial strain were summarized based on datasets: HM-276D Nanopore **(A)**, HM-276D PacBio **(B)** and HM-277D Nanopore **(C)** across ranks. Colors indicates different metrics: sensitivity, precision and accuracy. Taxon were accurately recovered above the family level. HM-276D Nanopore outperformed other two data sets. AB, *Acinetobacter baumannii*; AO, *Actinomyces odontolyticus*; BC, *Bacillus cereus*; BV, *Bacteroides vulgatus*; CB, *Clostridium beijerinckii*; DR, *Deinococcus radiodurans*; DF, *Enterococcus faecalis*; EC, *Escherichia coli*; HP, *Helicobacter pylori*; LG, *Lactobacillus gasseri*; LM, *Listeria monocytogenes*; NM, *Neisseria meningitides*; PAN, *Propionibacterium acnes*; PAG, *Pseudomonas aeruginosa*; RS, *Rhodobacter sphaeroides*; SAR, *Staphylococcus aureus*; SE, *Staphylococcus epidermidis*; SAL, *Streptococcus agalactiae*; SM, *Streptococcus mutans*; SP, *Streptococcus pneumonia*.

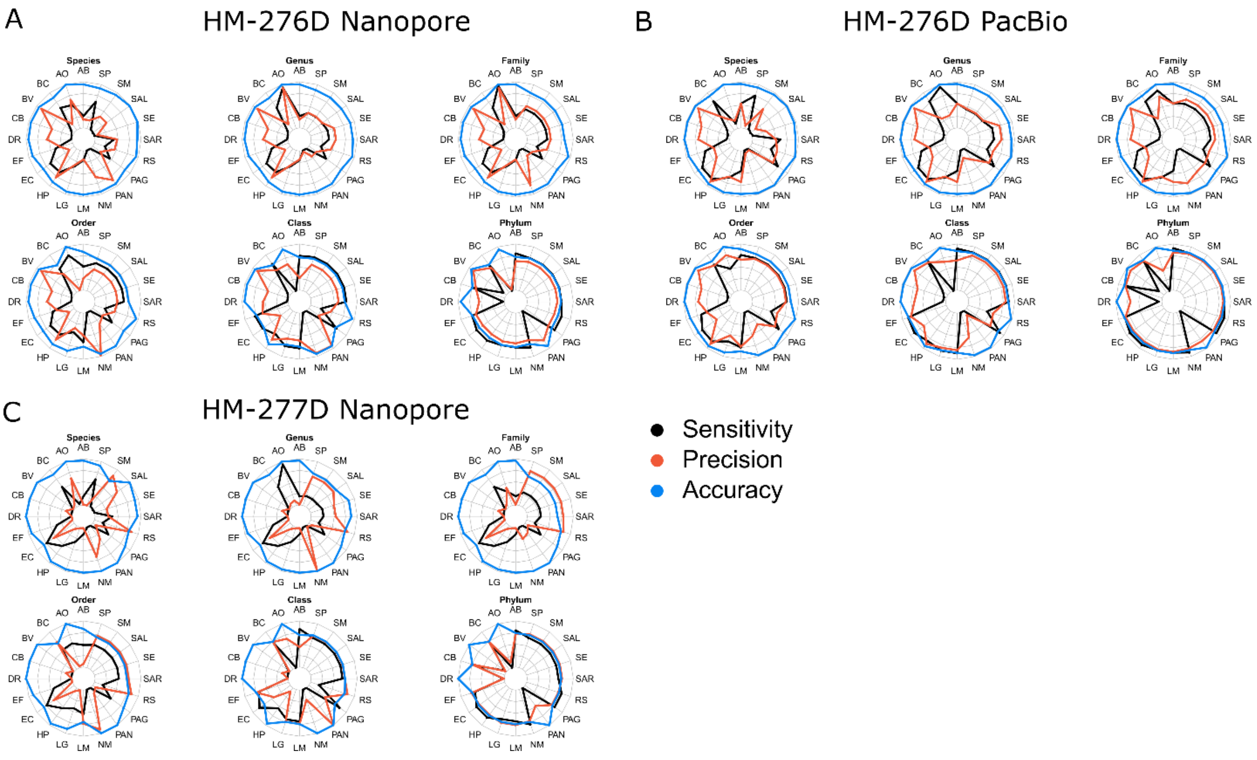

Figure S12. Strain-specific base pair assignment performance comparison across sequencing technologies, Related to Figure 4. Read base assignment accuracy statistics for each bacterial strain were summarized based on datasets: HM-276D Nanopore **(A)**, HM-276D PacBio **(B)** and HM-277D Nanopore **(C)** across ranks. Colors indicates different metrics: sensitivity, precision and accuracy. PacBio performed better than Nanopore data above the family level because of lower error rate. AB, *Acinetobacter baumannii*; AO, *Actinomyces odontolyticus*; BC, *Bacillus cereus*; BV, *Bacteroides vulgatus*; CB, *Clostridium beijerinckii*; DR, *Deinococcus radiodurans*; DF, *Enterococcus faecalis*; EC, *Escherichia coli*; HP, *Helicobacter pylori*; LG, *Lactobacillus gasseri*; LM, *Listeria monocytogenes*; NM, *Neisseria meningitides*; PAN, *Propionibacterium acnes*; PAG, *Pseudomonas aeruginosa*; RS, *Rhodobacter sphaeroides*; SAR, *Staphylococcus aureus*; SE, *Staphylococcus epidermidis*; SAL, *Streptococcus agalactiae*; SM, *Streptococcus mutans*; SP, *Streptococcus pneumonia*.

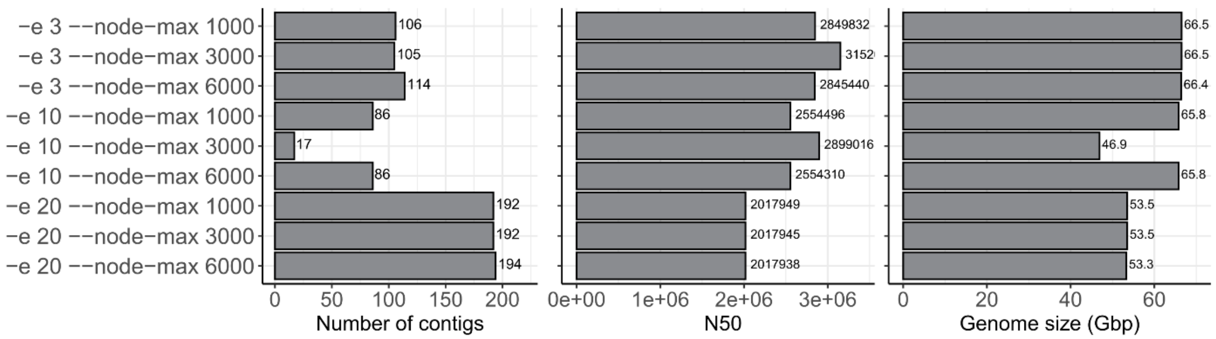

Figure S13. Assembly results for wtdbg2 based on HM-276D data sets, Related to Figure 2. Barplots indicate assembly statistics (Number of contigs, N50 length and genome size). Each row represents a run of wtdbg2 with parameters "-e" and "--node-max".

Tools	Depth	N50 length	NG50 length	NA50 length	NGA50 length	Accuracy (%)	Fraction (%)	# contigs	# long contigs	Longest contig	Genome size	CPU time (min)
**Canu**	**20x**	717267	616530	450953	413727	98.5	96.8	298	254	2612567	65503873	98
**Canu**	**40x**	1987236	1975600	893966	893966	99.07	99.29	132	112	6286130	67676017	250
**Canu**	**80x**	2886059	2731942	1284219	1284219	99.24	99.86	62	57	6316623	68735511	678
**Canu**	**160x**	3901381	3901381	1302124	1506778	99.27	99.93	60	52	6299115	68879111	1537
**Canu**	**365x**	2983818	2983818	1219953	1219953	99.28	99.83	64	58	6292103	68964121	2651
**Canu**	**480x**	3911963	3911963	1400993	1400993	99.4	99.81	83	65	6359094	69425747	3858
**OPERA-MS**	**20x**	1122204	1122204	450323	489324	99.83	99.71	5117	201	6324007	67168904	61
**OPERA-MS**	**40x**	2657727	2657727	1210958	1210958	99.96	99.99	1695	81	5220208	67629371	102
**OPERA-MS**	**80x**	2835709	2732545	1189226	1189226	99.96	99.99	1921	74	4636570	67632885	186
**OPERA-MS**	**160x**	2933262	2792941	1273171	1273171	99.95	98.45	2347	65	6255842	66580943	382
**OPERA-MS**	**365x**	2938016	2938016	1298425	1298425	99.91	99.98	4734	64	6255878	67858470	856
**OPERA-MS**	**480x**	2938019	2938019	1213537	1298125	99.92	99.98	4732	63	6255756	67892051	1238
**wtdbg2**	**20x**	552100	415407	243026	208370	96.22	90.73	439	367	3542441	60910472	3
**wtdbg2**	**40x**	2106610	2057746	751004	751004	98.1	98.58	143	110	6265362	66286613	7
**wtdbg2**	**80x**	3152112	2920474	1331021	1331021	98.79	98.97	105	66	6229939	66511311	13
**wtdbg2**	**160x**	2910424	2910424	1286826	1286826	98.95	98.75	134	76	6215258	66615029	13
**wtdbg2**	**365x**	2706821	2706821	1278483	1265683	98.66	97.34	90	73	6251621	65544245	19
**wtdbg2**	**480x**	3168384	2922530	1201346	1184259	98.73	95.95	201	119	6210210	65977641	23
**meta-flye**	**20x**	1653589	1547909	558652	534341	98.96	98.76	223	206	5630982	66808399	57
**meta-flye**	**40x**	2725547	2653197	1209147	1209147	99.43	99.97	64	52	6274273	67627825	86
**meta-flye**	**80x**	2930772	2930772	1588493	1588493	99.52	99.99	59	43	6251934	67630110	140
**meta-flye**	**160x**	3888260	3180529	1622636	1622636	99.54	99.97	61	39	6252579	67595608	372
**meta-flye**	**365x**	3181836	2934283	1315358	1315358	99.62	99.98	88	44	6245780	67727067	603
**meta-flye**	**480x**	3181822	2934277	1718698	1718698	99.62	99.99	89	43	6245565	67700317	756

Table S1. Comprehensive assembly statistics on HM-276D using Canu, OPERA-MS, wtdbg2 and meta-flye, Related to Figure 2.

Species	Abundance	RefSeq gene		16S rRNA gene		Protein coding gene	
		average coverage (#bases)	Significantly detected gene	average coverage (#bases)	Significantly detected gene	average coverage (#bases)	Significantly detected gene
**Acinetobacter baumannii**	0.18%	9.83	94	9.50	6	9.86	3,817
**Actinomyces odontolyticus**	0.01%	4.27	56	3.10	2	4.65	1,999
**Bacillus cereus**	1.22%	100.51	138	94.04	12	102.33	5,675
**Bacteroides vulgatus**	0.02%	2.32	65	1.77	4	2.39	3,067
**Clostridium beijerinckii**	1.43%	96.40	143	78.49	14	97.42	5,149
**Deinococcus radiodurans**	0.03%	4.94	57	5.19	3	4.86	3,060
**Enterococcus faecalis**	0.01%	2.76	53	3.81	2	3.37	2,497
**Escherichia coli**	15.75%	1,032.93	179	1,003.79	7	1,060.46	4,341
**Helicobacter pylori**	0.07%	113.13	43	117.15	2	114.16	1,444
**Lactobacillus gasseri**	0.03%	27.95	96	24.06	6	28.97	1,783
**Listeria monocytogenes**	0.07%	10.74	184	8.92	6	11.42	2,864
**Neisseria meningitides**	0.07%	42.67	71	28.53	4	47.85	1,926
**Propionibacterium acnes**	0.11%	41.60	58	38.75	3	43.02	2,506
**Pseudomonas aeruginosa**	5.01%	141.55	105	160.86	4	137.90	5,572
**Rhodobacter sphaeroides**	64.44%	2,219.40	67	1,993.22	3	2,438.52	4,279
**Staphylococcus aureus**	0.83%	323.26	79	289.00	5	404.68	2,982
**Staphylococcus epidermidis**	6.52%	976.37	76	1,117.10	5	1,002.43	2,472
**Streptococcus agalactiae**	0.03%	72.99	101	70.16	7	75.54	2,127
**Streptococcus mutans**	4.15%	4,207.60	80	3,598.02	5	3,818.93	1,953
**Streptococcus pneumoniae**	0.01%	1.91	58	1.30	2	2.39	1,868

Table S2. Species-specific gene coverage summary of HM-277D data set, Related to Figure 4. Gene coverage statistics were summarized for 3 different gene sets: all Refseq genes, 16S rRNA genes and protein coding genes. Average coverage = number of bases mapped to the exonic region / length of exonic region. Gene is noted as

significantly detected when 50% exonic region is covered by at least 1 read and average coverage > 1.

Transparent Methods

Oxford nanopore sequencing of HM-276D and HM-277D

DNA samples of HM-276D and HM-277D were ordered from BEI Resources. Concentration of DNA was assessed using the dsDNA HS assay on a Qubit fluorometer (Thermo Fisher).

For library preparation, 1.0 µg DNA was used as the input DNA of each library. The library was prepared using the ligation sequencing protocol (SQK-LSK109) from ONT. Concretely, end repair, dA-tailing and DNA repair was performed using NEBNext Ultra II End Repair/dA-tailing Module (catalog No. E7546) and NEBNext FFPE Repair Mix (M6630). In all, 3.5 µl Ultra II End-prep reaction buffer, 3 µl Ultra II End-prep enzyme mix, 3.5 µl NEBNext FFPE DNA Repair Buffer and 2 µl NEBNext FFPE DNA Repair Mix were added to the input DNA. The total volume was adjusted to 60 µl by adding nuclease-free water (NFW). The mixture was incubated at 20 °C for 5 min and 65 °C for 5 min. A 1 × volume (60 µl) AMPure XP clean-up was performed and the DNA was eluted in 61 µl NFW. One microliter of the eluted dA-tailed DNA was quantified using the Qubit fluorometer. A total of 0.7 µg DNA should be retained if the process is successful.

Adaptor ligation was performed using the following steps. Five microliter Adaptor Mix (ONT, SQK-LSK109 Kit), 25 µl Ligation Buffer (ONT, SQK-LSK109 Kit) and 10 µl NEBNext Quick T4 DNA Ligase (NEB, catalog No. E6056) were added to the 60 µl dA-tailed DNA from the previous step. The mixture was incubated at room temperature for 10 min. The adaptor-ligated DNA was cleaned up using 40 µl AMPure XP beads. The mixture of DNA and AMPure XP beads was incubated for 5 min at room temperature and the pellet was washed twice by 250 µl Long Fragment Buffer (ONT, SQK-LSK109). The purified-ligated DNA was resuspended in 15 µl Elution Buffer (ONT, SQK-LSK109). A 1-µl aliquot was quantified by fluorometry (Qubit) to ensure ≥ 400 ng DNA was retained.

The final library was prepared by mixing 37.5 µl Sequencing Buffer (ONT, SQK-LSK109), 25.5 µl Loading Beads (ONT, SQK-LSK109), and 12 µl purified-ligated DNA. The library was loaded to R9.4 flow cells (FLO-MIN106, ONT) according to the manufacturer's guidelines. GridION sequencing was performed using default settings for the R9.4 flow cell and SQK-LSK109 library preparation kit. The sequencing was controlled and monitored using the MinKNOW software developed by ONT.

Metagenome assembly

Genome assemblies of the 20-mixed bacteria from HM-276D and MH-277D mock communities were conducted using 4 existing assemblers based on generated long-read sequencing reads. These 4 dedicated long-read assemblers we used are wtdbg2 (v2.4), OPERA-MS, Canu (v1.8) and meta-flye, where OPERA-MS and meta-flye are designed to be capable to handle metagenome while wdtbg2 and Canu are for broadly application. To evaluate the impact of coverage depth in genome assembly, in addition to 525× (HM-276D) and 1068× (HM-277D), we subsampled 5 data sets with 365×, 160×, 80×, 40× and 20× coverages for these two mock communities. In addition to long-read data, OPERA-MS requires short reads to improve the assembly accuracy. Hence, we downloaded Illumina sequenced HM-276D(Jones et al., 2015) and HM-277D data sets(Kuleshov et al., 2016). Similarly, these short-read data were also subsampled with depths 160×, 80×, 40× and 20×, which were provided to OPERA-MS in corresponding data set analysis. We also analyzed a PacBio data set(Lee et al., 2014) of HM-276D sample using wtdbg2, OPERA-MS, Canu and meta-flye to compare assembly performance across sequencing technologies. For comparison fairness, we applied consistent configuration settings for each tool across different coverage depths. For wtdbg2, we first tuned parameters on "-e", "--node-max". Assemblies were conducted under different parameter values (-e: 3, 10, 20) (--node-max: 1000, 3000, 6000). Based on the genome contiguity and completeness results in **Fig S13**, we specified parameter "-e 3 –node-max" for wtdbg2. For other tools, we set estimated genome size as 70M, where the parameters are "genomeSize=70M useGrid=True" for Canu, and "CONTIG_LEN_THR 500, CONTIG_EDGE_LEN 80, CONTIG_WINDOW_LEN 340, KMER_SIZE 60, LONG_READ_MAPPER minimap2" for OPERA-MS, "-t 40 -g 70m -o ./ --meta" for meta-flye. 40 contig output files were obtained

(2 mock community samples, 6 depths of coverage, 4 assembly tools) for further evaluation.

Metagenome assembly evaluation

Assembled genomes produced by each tool based on different samples and coverage depths were evaluated with metrics related to contiguity, genome completeness and accuracy. To assess the assembly contiguity, we first used our script to calculate the widely-used statistic N50, which is the shortest contig needed to cover at least 50% of the assembly. In addition, other related statistics, such as number of contigs, number of long contigs (>10kb), longest contigs and total assembly size, were collected from the FASTA output file of each assembler. Furthermore, we summarized NG50 for each method by replacing the assembly size with estimated genome size. This quantity represents the shortest contig needed to cover 50% of the genome. Instead of contigs, we also evaluated the performance based on aligned blocks for each method by using QUAST(Gurevich et al., 2013) to calculate NA50 and NGA50, which represent shortest aligned block to cover 50% of the assembly and genome respectively. Based on these metrics, the contiguity of assemblies was comprehensively evaluated. Next, we downloaded the reference genome FASTA files of all 20 bacteria from NCBI database to measure the concordance between the references and assemblies. First, assemblies were mapped to the reference genomes using Mummer v3.23 with parameters “-maxmatch -c 100 -p nucmer”. Then, by comparing all contigs mapped onto the reference using dandiff, assembly accuracy was calculated using 1-to-1 alignment identity, which is the correctly matched base-pair percentage of contigs uniquely mapped to the reference genome (1-mismatch%). In addition, to assess the assembly completeness, we calculated the percentage of genome covered by the contigs. In real case, instead of evenly mixed in HM-276D mock community, bacterial strains are non-uniformly distributed, where some are likely to share extremely low abundance. Therefore, we evaluated the impact of the genomic DNA abundance on genome assembly. For the unevenly mixed HM-277D mock community samples, we calculated the abundance for each bacterial strain by normalizing the concentration with related reference genome size. The relationship between abundances

and assessment metrics was displayed using scatter plots. For each plot, linearity was measured based on Spearman correlation using R v3.3.3.

Taxonomic binning analysis

Taxon bins of the 20-mixed bacteria from two mock communities were recovered using taxonomic binner Megan-LR(Huson et al., 2018) with 3 long-read sequencing data sets: HM-276D (Nanopore, PacBio) and HM-277D (Nanopore) at 160× depth of coverage. We first aligned all reads against NCBI-nr protein reference database using LAST with parameters "-P 100 -F15". Next, output MAF files were converted to DAA format in smaller size. Then, we meganized the DAA files using MEGAN(Huson et al., 2016), which allows us to interactively visualize and explore these taxonomic results. To evaluate the taxonomic binning performance, we first counted the number of reads and bases which were correctly assigned to each taxon from the mock microbial community. We determined the metrics (precision, sensitivity, true positive rate and false positive rate). Precision and sensitivity assess how accuracy each read is classified across different sequencing technologies. Precision is the percentage of reads assigned correctly to the corresponding taxa out of all reads. Sensitivity is the percentage of correct reads out of reads assigned to the particular taxa. Next, we use true positive rate (TPR) and false discover rate (FDR) to assess the accuracy in taxonomic detection across sequencing technologies. TPR is the percentage of correctly detected taxon out of known taxon from the microbial community. FDR is the percentage of correctly detected taxon out of all detected taxon. All metrics are defined at each taxonomic rank.

Supplemental References

GUREVICH, A., SAVELIEV, V., VYAHHI, N. & TESLER, G. 2013. QUAST: quality assessment tool for genome assemblies. *Bioinformatics,* 29**,** 1072-5.

HUSON, D. H., ALBRECHT, B., BAGCI, C., BESSARAB, I., GORSKA, A., JOLIC, D. & WILLIAMS, R. B. H. 2018. MEGAN-LR: new algorithms allow accurate binning and easy interactive exploration of metagenomic long reads and contigs. *Biol Direct,* 13**,** 6.

HUSON, D. H., BEIER, S., FLADE, I., GORSKA, A., EL-HADIDI, M., MITRA, S., RUSCHEWEYH, H. J. & TAPPU, R. 2016. MEGAN Community Edition - Interactive Exploration and Analysis of Large-Scale Microbiome Sequencing Data. *PLoS Comput Biol,* 12**,** e1004957.

JONES, M. B., HIGHLANDER, S. K., ANDERSON, E. L., LI, W., DAYRIT, M., KLITGORD, N., FABANI, M. M., SEGURITAN, V., GREEN, J., PRIDE, D. T., YOOSEPH, S., BIGGS, W., NELSON, K. E. & VENTER, J. C. 2015. Library preparation methodology can influence genomic and functional predictions in human microbiome research. *Proc Natl Acad Sci U S A,* 112**,** 14024-9.

KULESHOV, V., JIANG, C., ZHOU, W., JAHANBANI, F., BATZOGLOU, S. & SNYDER, M. 2016. Synthetic long-read sequencing reveals intraspecies diversity in the human microbiome. *Nat Biotechnol,* 34**,** 64-9.

LEE, C. H., BOWMAN, B. & HALL, R. Developments in PacBio® metagenome sequencing: Shotgun whole genomes and full-length 16S. International Plant and Animal Genome Conference Asia, 2014.
